# Rapid diagnostic assay for detection of cellulose in urine as biomarker for biofilm-related urinary tract infections

**DOI:** 10.1038/s41522-018-0069-y

**Published:** 2018-10-26

**Authors:** Haris Antypas, Ferdinand X. Choong, Ben Libberton, Annelie Brauner, Agneta Richter-Dahlfors

**Affiliations:** 1 Department of Neuroscience, Swedish Medical Nanoscience Center, Karolinska Institutet, Stockholm, Sweden; 2 Department of Microbiology, Tumor and Cell Biology, Karolinska Institutet, Stockholm, Sweden; 3 Division of Clinical Microbiology, Karolinska University Hospital, Stockholm, Sweden; 4 Present Address: MAX IV Laboratory, Lund University, Lund, Sweden

## Abstract

The ability of uropathogenic *Escherichia coli* (UPEC) to adopt a biofilm lifestyle in the urinary tract is suggested as one cause of recurrent urinary tract infections (UTIs). A clinical role of UPEC biofilm is further supported by the presence of bacterial aggregates in urine of UTI patients. Yet, no diagnostics exist to differentiate between the planktonic and biofilm lifestyle of bacteria. Here, we developed a rapid diagnostic assay for biofilm-related UTI, based on the detection of cellulose in urine. Cellulose, a component of biofilm extracellular matrix, is detected by a luminescent-conjugated oligothiophene, which emits a conformation-dependent fluorescence spectrum when bound to a target molecule. We first defined the cellulose-specific spectral signature in the extracellular matrix of UPEC biofilm colonies, and used these settings to detect cellulose in urine. To translate this optotracing assay for clinical use, we composed a workflow that enabled rapid isolation of urine sediment and screening for the presence of UPEC-derived cellulose in <45 min. Using multivariate analysis, we analyzed spectral information obtained between 464 and 508 nm by optotracing of urine from 182 UTI patients and 8 healthy volunteers. Cellulose was detected in 14.8% of UTI urine samples. Using cellulose as a biomarker for biofilm-related UTI, our data provide direct evidence that UPEC forms biofilm in the urinary tract. Clinical implementation of this rapid, non-invasive and user-friendly optotracing diagnostic assay will potentially aid clinicians in the design of effective antibiotic treatment.

## Urinary tract infections: Lighting up the biofilm state

A rapid test for the presence of bacterial cellulose in urine allows urinary tract infections due to bacteria in the biofilm state to be distinguished from less problematic ‘planktonic’ infections caused by free-living bacteria. Agneta Richter-Dahlfors and colleagues at the Karolinska Institute in Stockholm, Sweden, developed the test based on the fluorescence of oligothiophene molecules when they bind to biofilm cellulose in urine. The researchers tested the procedure using ‘uropathogenic *Escherichia coli*‘ (UPEC) infections in patients. The results also confirmed that UPEC infections can indeed form biofilms in the urinary tract. Previously, there has been no way to readily distinguish between urinary tract infections caused by bacteria in the free-living and biofilm states. This non-invasive and simple procedure could assist clinicians in selecting the most appropriate treatment for an infection.

## Introduction

Biofilms are linked with chronic and recurrent infections that are resistant to treatments and hard to eradicate.^1^ In biofilm-related infections, the microbial community can be found directly associated with a patient’s tissue or with foreign bodies, such as medical devices or implants. Biofilms are defined by the presence of bacterial aggregates embedded in a self-produced extracellular matrix (ECM) composed of extracellular polymeric substances (EPS).^2^ The tolerance to antibiotic treatment is mainly attributed to the distinct physiology bacteria adopt within a biofilm, but also to the specific microenvironment of the infection site.^3^ The metabolic heterogeneity of bacteria and the complex structure of a biofilm, combined with the hypoxic environment at the infection site, often accounts for the ineffectiveness of otherwise clinically relevant antibiotic treatments.^4–10^

Patients with urinary tract infection (UTI) often suffer from recurrent infections,^11^ which may be attributed to biofilms.^11–13^ Uropathogenic *Escherichia coli* (UPEC), the major causative agent of UTI, produces biofilm *in vitro* with the polysaccharide cellulose and amyloid protein curli as the major EPS.^14,15^ Studies in animal models have shown that UPEC most likely forms biofilm to facilitate colonization of the renal proximal tubule.^16^ Intracellular bacterial communities forming biofilm-like aggregates have been observed on the prostate glands,^17^ within the superficial cells of the bladder^18^ as well as in urine from UTI patients.^19,20^ Despite this evidence, no clinically established methods exist that provide a definitive diagnosis of biofilm-related UTI.

In routine clinical diagnostics of UTI, culture-dependent testing is based on planktonically growing cultures. While this approach is well suited to identify bacterial species, it fails to define whether bacteria originally formed biofilm in the patient. Similarly, genotypic methods cannot discriminate between planktonic bacteria and a biofilm lifestyle.^21–23^ To date, microscopy-based methods are commonly used to detect bacterial aggregates directly in the patient samples. These aggregates, assumed to represent a biofilm, are visualized by non-specific dyes, such as the Gram stain, or by species-specific staining, exemplified by fluorescence *in situ* hybridization (FISH)^24^ and peptide nucleic acid FISH (PNA FISH), using fluorescence confocal microscopy.^25^ To visualize EPS, carbohydrate stains such as alcian blue, calcofluor, and ruthenium red are applied along with fluorescently labeled lectins.^26–29^ The interpretation of such analysis is, however, subjective as these stains are far from specific for biofilm components. In a limited number of cases, biofilm-specific EPS detection in patient samples has been achieved. Immunofluorescence microscopy using antibodies specific for the EPS alginate was used to detect *Pseudomonas aeruginosa* biofilms^24^ and immuno-electron microscopy using antibodies targeting the amyloid curli protein was used to detect UPEC biofilms.^15^ Despite specific reporting of biofilms, these advanced and labor-intensive techniques are of limited use for routine analysis in a clinical laboratory, where hundreds of samples can arrive daily.

We recently reported optotracing as a new method for real-time, *in situ* detection and differentiation of the *Salmonella* biofilm components curli and cellulose.^30^ Optotracing is based on a class of non-toxic molecules, the luminescent-conjugated oligothiophenes (LCOs), which are flexible conjugated polymers emitting conformation-dependent fluorescence spectra.^31^ Binding of the heptameric LCO heptamer formyl thiophene acetic acid (h-FTAA) to EPS molecules in *Salmonella* biofilm resulted in linearization of the oligothiophene backbone of h-FTAA, which was observed as a red shift of the excitation spectrum compared to that from unbound h-FTAA. The spectral signature from h-FTAA bound to cellulose shows unique peaks at 464 and 488 nm, which are characteristic of cellulose interaction. A detailed analysis of the molecular interaction between h-FTAA and the cellulose polysaccharide showed an exceptional specificity to β-1,4 configured glucans.^32^ This suggests a universal use of this optotracer for the detection of cellulose across biological kingdoms.

Cellulose production by UPEC serves as an indication that bacteria have entered the biofilm mode of growth.^33–36^ As cellulose is not naturally found within the human body, we hypothesized that any cellulose identifiable in the urine of UTI patients must have been expelled from UPEC biofilms in the urinary tract. Here, we investigated whether biofilm is formed in UTI patients by developing an assay for detection of cellulose in urine, and whether cellulose can be used as a candidate diagnostic biomarker for biofilm-related UTIs.

## Results

### Evaluation of cellulose optotracing directly in urine

**Fig. 1:**
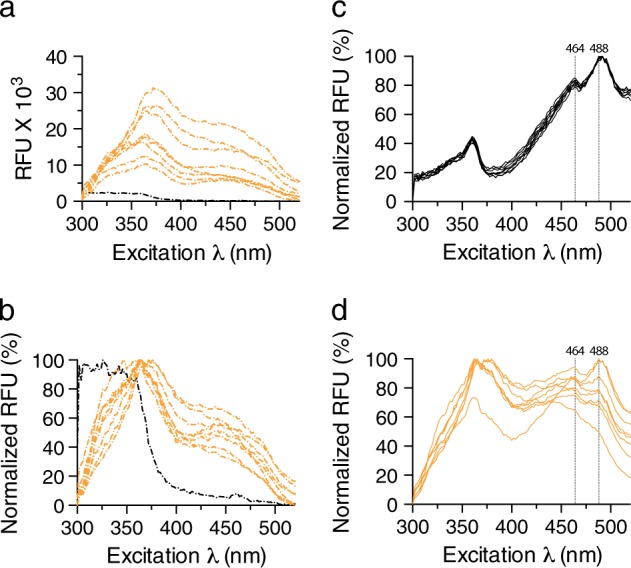
Optotracing for direct detection of cellulose in urine. **a** Excitation spectra and **b** normalized excitation spectra of urine from eight healthy volunteers (dash-dot orange) and PBS (dash-dot black) excited at 300–520 nm with emission collected at 545 nm. Average fluorescence of two technical replicates for each sample is shown. **c**, **d** Normalized excitation spectra showing optotracing of M. cellulose mixed with h-FTAA in **c** PBS and **d** healthy urine. Each line represents the average normalized fluorescence from three technical replicates per preparation. Dotted vertical lines = 464 and 488 nm representing the cellulose signature

Human urine represents a complex aqueous solution of metabolites with significant native fluorescence.^37^ Since this may interfere with optotracing, we started by analyzing urine’s fluorescence in the spectral range relevant for cellulose detection. To this end, we collected urine from eight healthy volunteers. Dipstick testing in these samples showed negative results for all UTI-associated parameters and urine cultures verified the absence of UPEC. Other bacterial species found were well below the limit for bacteriuria (Table 1). Following transfer of urine aliquots into 96-well plates, a plate reader was used to record excitation spectra of each sample at 300–520 nm with emission collected at 545 nm. Phosphate-buffered saline (PBS) was profiled alongside urine samples for comparison. Analysis of data in a spectral plot (spec-plot) revealed substantial native fluorescence across the full range of excitation wavelengths (Ex. λ) (Fig. 1a). Individual variations were observed, with maximal relative fluorescence units (RFU_max_) ranging between 9 and 31 × 10^3^. We further analyzed the data in a normalized spec-plot, by assigning 0 % to the lowest and 100 % to the highest RFU in each sample. This showed that the wavelengths corresponding to the maximum emitted fluorescence (Ex. λ_max_) were between 360 and 380 nm in all urine samples (Fig. 1b). In contrast to urine, PBS showed RFU_max_ as low as 2.5 × 10^3^, and this signal decreased even more at wavelengths longer than 380 nm (Fig. 1a, b). Overall, urine was predominantly fluorescent at the shorter wavelengths, which are of lesser importance during cellulose optotracing, as the cellulose spectral signature is expected at wavelengths longer than 400 nm.^30^ We concluded that cellulose optotracing ought to generate signals with enough fluorescent amplitude to be visible at wavelengths longer than 400 nm, despite urine’s native fluorescence.

**Table 1:** Characterization of urine from healthy volunteers

Sample #	Urine culture	
	Bacterial load (CFU/ml)	Organism
1	1 × 10^2^	*Enterococcus* spp.
2	0	NA^a^
3	2 × 10^3^	*Lactobacillus*
4	3 × 10^2^	*Staphylococcus* or *Streptococcus*
5	2 × 10^2^	*Staphylococcus* or *Streptococcus*
6	4 × 10^2^	*Staphylococcus* or *Streptococcus*
7	1 × 10^2^	*Enterococcus* spp.
8	1 × 10^2^	*Enterococcus* spp.

^a^Not applicable

Next, we identified the optical signatures of cellulose by performing optotracing with h-FTAA in eight preparations of 125 µg/ml microcrystalline cellulose (M. cellulose) in PBS. The normalized spec-plots showed great consistency as spectra from the eight preparations were superimposed on each other. Two distinct peaks were observed, one with Ex. λ_max_ at 488 nm and one with lower RFU at 464 nm (Fig. 1c). These two peaks define the cellulose optical signature and they are clear indicators of cellulose in the sample.^30^ To define the detection limit of our method, we made 2-fold dilution series of the M. cellulose preparations in PBS, and included PBS-only as blank. When performing optotracing, we obtained a clear spectral signature in all of the eight cellulose preparations containing 62.5 µg/ml (Fig. S1a). A majority of the samples, five out of eight preparations, also provided clear cellulose signatures at 31.3 µg/ml (Fig. S1b). In contrast, the cellulose signal was absent at lower concentrations (Fig. S1c, d), with some of the spectra mimicking the PBS blank (Fig. S1e). We thus conclude that the detection limit of optotracing for cellulose in PBS ranges between 31.3 and 62.5 µg/ml.

To test whether optotracing for cellulose also can be achieved in urine, we spiked the eight urine samples from healthy volunteers with 125 µg/ml each of M. cellulose, added h-FTAA, and collected the excitation spectra. We detected the cellulose signature in two of the samples, with Ex. λ_max_ at 488 nm and the minor peak at 464 nm (Fig. 1d). In the remaining samples, we observed only weak cellulose spectral signatures or none at all, suggesting that the cellulose optotracing signal is dominated by the emission of urine’s native fluorescence. To define the detection limit, we spiked the eight healthy urine samples with the M. cellulose, made 2-fold serial dilutions of each sample, and performed optotracing, using urine-only samples as blank. A clear cellulose signature was observed in two out of eight samples at 62.5 µg/ml (Fig. S1f), while spectra at all lower concentrations resembled samples containing urine only (Fig. S1g–j). Whereas these results demonstrate that cellulose optotracing directly in urine is feasible, native fluorescence is indeed a concern for the consistency of the detection limit, especially since we noted a great variability in the intrinsic fluorescence between individuals. Samples with high fluorescence are thus at risk of affecting optotracing’s detection sensitivity. Collectively, these results show that PBS is better suited for cellulose optotracing than urine, as consistent results are generated at a lower detection limit.

### Detection of cellulose in UPEC biofilms

**Fig. 2:**
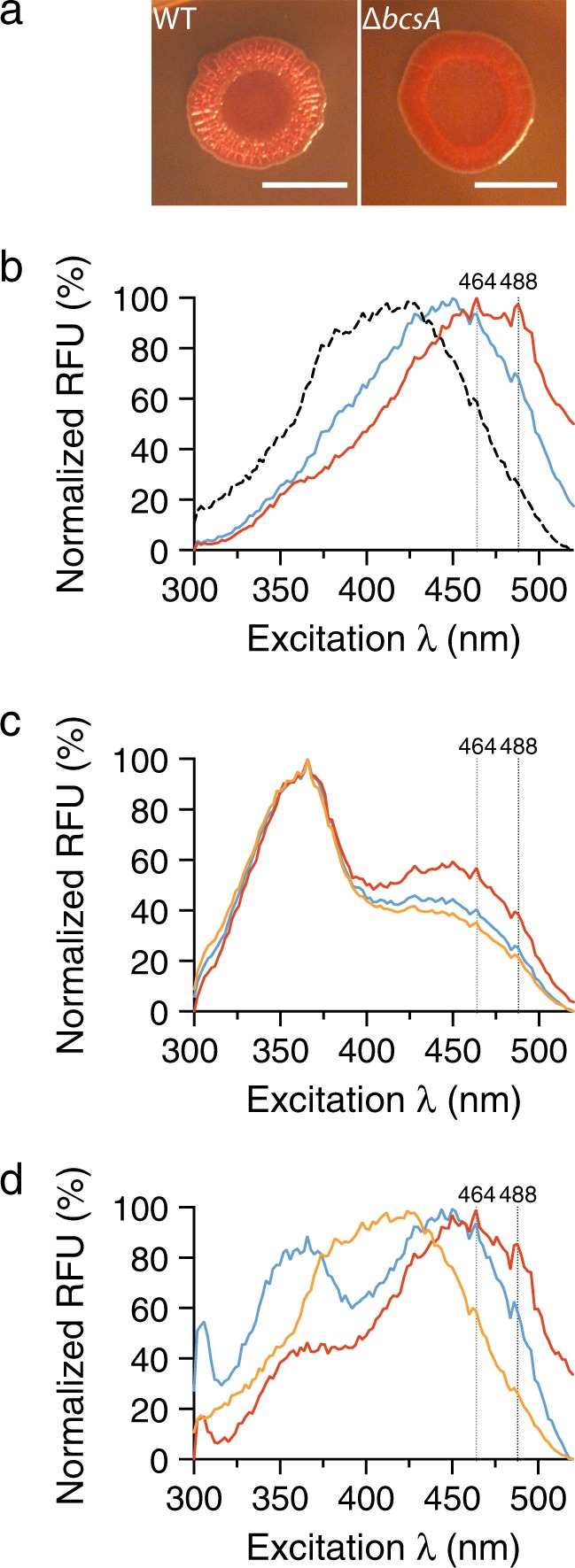
Cellulose detection in UPEC biofilms by optotracing. **a** Colony morphotypes of UPEC No. 12 WT and Δ*bcsA* on Congo red agar plates. Scale bar = 1 cm. **b** Normalized spec-plot of biofilms from UPEC No. 12 WT (red) and Δ*bcsA* (blue) resuspended in PBS containing h-FTAA, and of unbound h-FTAA in PBS (black). Average normalized fluorescence from three biological replicates is shown. **c** Normalized spec-plot of biofilms from UPEC No. 12 WT (red) and Δ*bcsA* (blue) resuspended in urine containing h-FTAA. Urine containing only h-FTAA (orange) was included for comparison. Average normalized fluorescence from three technical replicates from one representative experiment is shown. **d** Normalized spec-plot showing optotracing of samples presented in **c** after they were centrifuged at 15,700 × *g* and resuspended in 500 µl PBS. Average normalized fluorescence from three technical replicates from one representative experiment is shown. Dotted vertical lines = 464 and 488 nm representing the cellulose signature

We next determined if cellulose can be detected in its native complexity within UPEC biofilms by optotracing. We used UPEC No. 12, a clinical uropathogenic *E. coli* strain isolated from a child with pyelonephritis.^15^ This wild-type (WT) strain is known to form biofilm when grown on agar plates under osmotic stress at 37 °C. In the presence of Congo red, the characteristic colony morphology termed *rdar* (*r*ed, *d*ry, *a*nd *r*ough) forms, indicating the presence of cellulose and curli in the biofilm’s ECM (Fig. 2a, WT). The isogenic strain UPEC No. 12 Δ*bcsA*, which has a mutation in the gene encoding the cellulose synthase catalytic subunit, renders this strain unable to produce cellulose. This is manifested by an inability to form the *rdar* morphotype (Fig. 2a, Δ*bcsA*). To perform optotracing for cellulose in UPEC biofilms, we harvested biofilm colonies from WT and Δ*bcsA* strains, resuspended them in PBS containing h-FTAA, and recorded the excitation spectra. Optotracing of the WT showed two peaks in the normalized spec-plot, both red-shifted compared to unbound h-FTAA (Fig. 2b). The appearance of Ex. λ_max_ at 464 nm and a minor peak at 488 nm, compared to Ex. λ_max_ at 400 nm of the unbound h-FTAA, strongly indicates the presence of cellulose. The spectrum of the mutant Δ*bcsA* lacked both peaks, which confirms the absence of cellulose in the sample, as expected from the genotype of this strain. The Ex. λ_max_ at 450 nm of the mutant strain results from binding of the optotracer to unknown components in the complex ECM.

To investigate whether we can detect cellulose from UPEC biofilm in urine, we spiked one aliquot of healthy urine containing h-FTAA with biofilm from the WT strain, and another with biofilm from the mutant Δ*bcsA*. As negative control, we used urine to which only h-FTAA was added. Analysis of the normalized spec-plot showed little differences between spiked and unspiked urine. Signals from the native fluorescence of urine dominated the three spectra, shown by Ex. λ_max_ at 360–380 nm (Fig. 2c). A marginally higher fluorescence in the range 425–500 nm was observed in urine spiked with biofilm from WT bacteria compared to biofilm from the mutant Δ*bcsA* and the negative control. Signals were thus not sufficient to consitute a cellulose signature.

To improve the sensitivity of optotracing for cellulose in native biofilms, we developed an alternative workflow. Knowing that optotracing generates very consistent signals for M. cellulose in PBS (Fig. 1c), we transferred bacteria and their biofilm components from urine to PBS before optotracing. A small volume of urine (1 ml) spiked with biofilm from the WT or the mutant Δ*bcsA*, as well as urine only (negative control), were centrifuged for 15 min. Pellets were resuspended in 0.5 ml PBS containing h-FTAA and then optotracing for cellulose was performed. The normalized spec-plot showed a distinctly red-shifted spectrum for the WT compared to the negative control, the cellulose-specific Ex. λ_max_ at 464 nm and the accessory peak at 488 nm (Fig. 2d). In contrast, biofilm from the mutant Δ*bcsA* showed Ex. λ_max_ at 450 nm with no indications of cellulose in the sample. Collectively, this shows that the transfer of biofilm from urine to PBS increases sensitivity for cellulose detection by optotracing.

### Cellulose as a biomarker for biofilm-associated UTIs

**Fig. 3:**
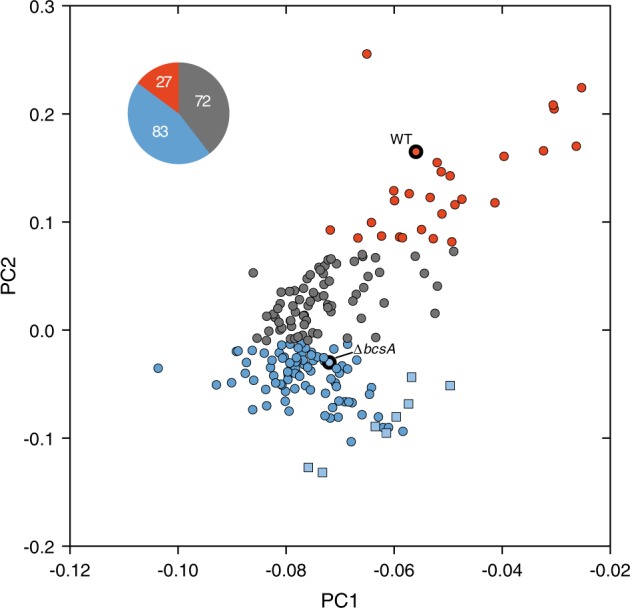
Optotracing of cellulose in urine from UTI patients. Principal component analysis (PCA) plot of cellulose optotracing in 182 urine samples from UTI patients (circle), eight healthy urine samples (square) and two biofilm preparations from UPEC No. 12 WT (thick border red circle) and Δ*bcsA* (thick border blue circle). Data from normalized excitation spectra in the cellulose signature wavelength range of 464–508 nm were analyzed with PCA and clustered with *k*-means clustering. The three generated clusters differentiate samples that are positive (red) and negative (blue) for cellulose, as well as samples with insufficient discriminatory performance (gray). The pie chart inset shows the number of UTI samples in each of the three clusters

Despite evidence of biofilm-like bacterial aggregates in urine from UTI patients, biofilm EPS, such as cellulose, have never been detected. Based on our rapid and user-friendly workflow for urine analysis, we investigated whether optotracing could be applied to detect cellulose in clinical urine samples. Urine samples from 182 patients, clinically diagnosed with UTI caused by UPEC, were collected. For comparative purposes, we included urine from the eight healthy volunteers, as well as biofilm samples from UPEC No. 12 WT and the mutant Δ*bcsA*, which served as cellulose-positive (WT) and cellulose-negative (Δ*bcsA*) references in the analysis. We isolated the sediments from all urine samples and resuspended them in PBS. By performing optotracing, we obtained spectral information from all 192 samples. To enable unbiased analysis of the spectral data and easy comparison of the multiple fine spectral details represented by the varying fluorescence intensity at each nanometer, we used principal component analysis (PCA). This type of analysis was applied to spectral information obtained between 464 and 508 nm, where the cellulose optical signature is expected. Principal component 1 (PC1) and principal component 2 (PC2) were selected, as they were found to account for >99% of the variation across excitation spectra from all strains. We applied *k*-means clustering to organize the spread of samples across PC1 and PC2, and the Elbow method to define the optimal number of clusters present in our PCA. Based on the similarities among excitation spectra, this analysis grouped samples into three clusters, which we colored red, gray, and blue in the PCA plot (Fig. 3).

The red cluster at the top right of the PCA plot included 27 UTI samples and the cellulose-positive reference (WT) (Fig. 3). Since this suggested the presence of cellulose, we selected representative UTI samples based on their distribution across this cluster for further analysis. UTI samples located to the top right (see nos. 66, 87, 146, 73 in the magnified red cluster in Figure S2a) showed Ex. λ_max_ at 488 nm and a smaller peak at 464 nm (Fig. S2b). This pattern overlapped with the WT strain, and resembled the typical signature of M. cellulose (Fig. 1c), suggesting a substantial abundance of cellulose in these samples. UTI samples spreading towards the bottom left (nos. 18, 65, 28, 17) showed Ex. λ_max_ at 464 nm, and an accompanying smaller peak at 488 nm (Fig. S2b). Overall, these data showed that cellulose is present in UTI samples. PCA and *k*-means clustering on defined and unknown samples enabled us to rapidly and accurately identify those containing cellulose. The altered position of Ex. λ_max_ between 464 and 488 nm observed in the spectra of this cluster may reflect differences in the amount of cellulose and the biofilm complexity in each sample.

The blue cluster at the lower end of the PCA plot includes 83 UTI samples and the cellulose-negative reference (Δ*bcsA*), (Fig. 3). As this suggested that these samples lacked cellulose, we selected representative samples across the full distribution of the blue cluster for further evaluation (Fig. S3a). Indeed, the normalized spec-plots from each selected UTI sample were similar with the cellulose-negative reference sample (Fig. S3b). This suggested that a majority of urine samples in our UTI collection does not contain cellulose.

The urine samples from our eight healthy volunteers were also located in the blue cluster, albeit relatively isolated in the periphery of the cluster (Fig. 3 and S3a). The normalized spec-plots of these samples (nos. 1–8) showed no presence of cellulose (Fig. S3c). Their distinct position at the periphery of the cluster showed that the content of healthy urine markedly differs from that of samples from patients with UTI. Thus, optotracing seems to differentiate healthy from infected urine.

The gray cluster in the central part of the PCA plot contains 72 UTI samples (Fig. 3). Close inspection of this cluster in a magnified plot showed an even sample distribution in the area between the red and blue cluster (Fig. S4a). The normalized spec-plot of samples closest to the red cluster (nos. 23, 57, 38) showed Ex. λ_max_ at 464 nm and a low-amplitude spike at 488 nm (Fig. S4b), suggesting the presence of cellulose. Analysis of samples located in the central area of the gray cluster (nos. 134, 69, 68) showed red-shifted Ex. λ_max_ compared to the cellulose-negative control Δ*bcsA*. However, lack of cellulose-specific peaks at 464 and 488 nm suggests an absence of cellulose in these samples. Finally, analysis of samples located close to the blue cluster (nos. 85, 110) showed Ex. λ_max_ at wavelengths shorter than 450 nm (Fig. S4b). The lack of peaks in the cellulose-specific region in most of the gray cluster samples suggests the absence of cellulose. Yet, these spectra differed from that of the cellulose-negative reference. We thus concluded that the discriminatory performance of the spectra generated from samples in the gray cluster was insufficient to determine the presence or absence of cellulose.

Taken together, our results support the application of optotracing combined with PCA for rapid screening and detection of cellulose in urine samples, as a biomarker for biofilm-related UTI. This analysis showed that cellulose-based biofilms are present in 14.8% of urine samples from UTI patients.

## Discussion

Biofilm is increasingly recognized as a main virulence factor in chronic and recurrent infections.^38^ Despite the progress made in understanding biofilm’s role in infection, development of diagnostic methods for biofilm presence is lagging behind. This is primarily due to lack of tools that detect biofilm-specific components. Here, we have developed a culture-independent assay that rapidly detects cellulose as a UPEC biofilm marker in urine from UTI patients. A urine sample of 1 ml is sufficient to isolate bacterial components and screen them for the presence of cellulose using the optotracing technology. Testing is based on standard instrumentation available in a clinical microbiology laboratory, and it is easily performed with a total hands-on time of less than an hour. To meet the clinical demand for rapid and high-throughput data analysis, we applied PCA and *k*-means clustering as a powerful, non-biased way to process results for interpretation. In this study, cellulose optotracing of 182 urine samples from patients with UTI indicated that cellulose was present in 14.8% of the patients. This proof of concept demonstrates that cellulose optotracing has great potential as a diagnostic method for biofilm-related infections.

Existing methods used for direct detection of biofilm in UTI samples are non-specific as they are based on detecting bacterial aggregates under microscopy, rather than specific EPS produced when bacteria enter a biofilm lifestyle.^24–29^ Interpretation of such results is subjective and does not provide a definitive way of diagnosing an infection as biofilm-associated. The core theory of the optotracing methodology applied here is the detection of a specified polysaccharide within a sample, based on a unique spectral signature. In this study, cellulose was the polysaccharide in focus as it is a major component of the ECM in UPEC biofilms. Owing to the ability of optotracing to specifically detect cellulose, we were able to determine whether a UTI was biofilm-associated.

Since the optotracer molecules bind directly to the polysaccharide backbone of cellulose, they can potentially be applied as universal detectors for cellulose produced by microbes since detection is independent of species-specific differences in cellulose morphology, density, and crystallinity.^32^ Furthermore, diagnostic optotracing of urine samples can be completed in less than an hour, owing to the simple sample preparation and the ability of optotracing to instantaneously generate spectral signatures upon cellulose binding.

Optotracing is a pioneering diagnostic assay for detection of native cellulose in urine. Central to its implementation in complex clinical samples is the non-disruptive nature of the method. All established methods involve hydrolysis of cellulose and detection of glucose, the monomeric unit of cellulose. Such methods are not suitable for cellulose diagnostics, as glucose in urine from UTI patients can originate from multiple host and bacterial sources. This made it difficult to establish a reference method to define the diagnostic accuracy of optotracing. We took, however, several measures to reduce the risk of misdiagnosing cellulose in samples. We used PBS as assay matrix to ensure low native fluorescence, increase the consistency of results during optotracing, and achieve a lower detection limit for cellulose. This minimized the risk of false-negative results due to the variability of urine among individuals. To increase the likelihood of detecting cellulose, we concentrated each sample by pelleting bacterial components. However, this also concentrated host components indiscriminately, such as red and white blood cells or exfoliated epithelial cells, into collected pellets. Although it is currently unknown whether host components would compromise optotracing, future improvements in sample pre-processing may increase detection sensitivity. It is also important to note that this method is specific for cellulose-expressing biofilms; infections caused by cellulose-expressing microbes other than UPEC would also give a positive result. Similarly, a negative result would not exclude a biofilm infection, as the EPS constituents may differ between strains. The risk of obtaining false-positive results is low. Cellulose is not naturally produced by humans, so its presence in urine ought to be of microbial origin. Bacteria other than UPEC found in low amounts in healthy urine samples (*Enterococcus*, *Lactobacillus*, *Staphylococcus*, *Streptococcus* spp.) did not generate any cellulose spectral signature. Among the species known to produce cellulose as a component of their biofilm ECM, only UPEC is of high clinical relevance for UTIs.^39^

We believe that a method for cellulose-based biofilm diagnostics would be of great clinical relevance. Information on biofilm diagnostics combined with species identification and antimicrobial susceptibility testing results from planktonic cultures could aid in the better design of antibiotic regimens. Although we are still far from establishing clinical breakpoints for biofilm-related infections, there is reported evidence for the differential effectiveness of antibiotics on biofilms. Some antibiotics have been shown to preferentially kill metabolically active bacteria in the outer layers of the biofilm, while others kill metabolically inactive bacteria in its inner part.^40–43^ The low oxygen levels in biofilms is another factor that affects the activity of antibiotics, such as Ciprofloxacin.^44^ Moreover, the biofilm matrix can trap enzymes, such as β-lactamases that hydrolyze β-lactam antibiotics.^45^ This means that the dose and the duration of therapy may have to be adjusted in the presence of biofilm and β-lactamase stable antibiotics, such as meropenem or imipenem, could be used instead. By knowing the presence of biofilm in a patient, the clinician could select a more effective panel of antibiotics and also attempt a combinational or sequential antibiotic therapy.^3^ Biofilm diagnostics could also help to evaluate alternative therapies to target biofilm.^38,46^ Finally, by establishing biofilm diagnostics in the clinics, a surveillance system can be implemented for patients with chronic and recurrent infections to evaluate the success of antibiotic treatment, or for catheterized patients at risk of developing catheter-associated infections.

In conclusion, the development and clinical testing of our method for cellulose diagnostics in biofilm UTI shows the potential of optotracing in clinical diagnostics. As the technology matures, we foresee an expansion of optotracing to polysaccharides and functional amyloids in ECM of other clinically significant microbial biofilms. When combined with multivariate analysis, optotracing showed promising discriminatory performance between healthy and infected urine samples. The relevance of this finding for clinical diagnostics remains to be seen. Ultimately, we envision optotracing of EPS as an easily integrable method alongside other routine diagnostics in the clinical laboratory that would improve diagnosis and treatment of biofilm infections.

## Methods

### Study design

Research was conducted in full conformance with the laws and regulation of Sweden. Collection and analysis of anonymous UTI urine samples was approved by Karolinska Institutet, Karolinska University Hospital and by Regionala etikprövningsnämnden (EPN), Stockholm (The Regional Ethics Committee, Stockholm), decision reference 2017/448-31/1. Since urine samples were collected anonymously, this study was exempted from the requirement of a written informed consent.

### Urine collection and characterization

Midstream urine from eight anonymous healthy volunteers was collected and immediately screened with a urine dipstick (Multistix® 10 SG reagent strip, Siemens, Germany). Urine cultures were performed by streaking 10 µl urine on Brilliance™ UTI Agar (Oxoid, UK), and incubating the plates at 37 °C. Next day, colony-forming units (CFUs) were counted, and bacterial species were identified based on the colony color. To investigate urine’s native fluorescence, 2 × 100 µl from each healthy urine sample were transferred in a transparent, flat-bottom 96-well plate, and excitation spectra of 300–520 nm with emission collected at 545 nm were collected using an Infinite® M1000 PRO plate reader (TECAN, Mannedorf, Switzerland).

Aliquots of 3–5 ml of urine samples were collected anonymously from 182 patients with confirmed UTI caused by >10^5^ CFU/ml of UPEC from Karolinska University Hospital, Stockholm, Sweden. All samples were immediately analyzed, and discarded after use.

### Biofilm cultivation and Congo red morphotyping

The clinical strain UPEC No. 12 (WT), isolated from a patient with UTI, and the mutant strain WE1 *bcsA*::Cm (Δ*bcsA*) were used as an isogenic pair of strains for controlled expression of cellulose.^15^ Single colonies of each strain were grown overnight in LB broth at 37 °C. The overnight cultures were washed twice in LB broth without sodium chloride (LB n.s.) and pellets were resuspended again in LB n.s. To grow biofilm colonies, 10 µl drops of each strain were applied on LB n.s. solid media, and plates were incubated for 48 h at 37 °C. For biofilm colony morphotyping, 10 µl drops were applied on Congo red solid media as previously described,^30^ and incubated for 48 h at 37 °C. Pictures of all morphotypes were taken.

### Cellulose optotracing

Optotracing for cellulose was performed using the LCO h-FTAA (gift from Prof. P. Nilsson, Linköping University, Linköping, Sweden).^47–49^ Stock solutions (1.5 mM) prepared in dH_2_O were maintained at 4 °C. From each sample preparation (see below), 50–150 μl were transferred in duplicates or triplicates to transparent, flat-bottom 96-well plates. h-FTAA was added to each well at a final concentration of 4.5 μM, and incubated for 15 min at room temperature. To collect excitation spectra, samples were excited between 300 and 520 nm using 2-nm steps and emission was collected at 545 nm in a plate reader.

To identify the cellulose detection limit, we prepared 2-fold dilution series of M. cellulose (Sigma-Aldrich, MO, USA), ranging from 125 to 7.8 µg/ml, in eight different healthy urine samples and in eight samples of PBS. We also prepared negative controls for each series with no cellulose. Samples were sonicated at 30% amplitude using a 2-mm sonicator probe (Sonics & Materials, CT, USA), following an on/off cycle of 5 s for a total of 40 s. To prepare biofilm for optotracing, single biofilm colonies from UPEC No. 12 WT and Δ*bcsA* were resuspended in 500 µl PBS or 1 ml of healthy urine. To transfer bacterial components from urine to PBS before optotracing, samples were centrifuged at 15,700 × *g* for 15 min at 4 °C and pellets were resuspended in fresh 500 µl PBS.

To prepare the 190 urine samples for optotracing, 1 ml of each sample was centrifuged at 15,700 × *g* for 15 min at 4 °C, and the urine sediment was resuspended in 500 µl PBS. Resuspensions were serially diluted 1:2, 1:4, and 1:8 in PBS before optotracing. Reference samples UPEC No. 12 WT and Δ*bcsA* were prepared by transferring single biofilm colonies into 500 μl PBS, which were vortexed vigorously for 10 s followed by centrifugation at 3300 × *g* for 15 s, and resuspension in 500 µl PBS. This washing step was repeated two more times. Washed biofilm samples were sonicated for 5 s at 30% amplitude on ice using a 2 mm sonicator probe (Sonics & Materials, CT, USA), pelleted at 3300 × *g* for 15 s, and resuspended in 500 μl PBS before optotracing was performed.

### Data analysis

Data from excitation spectra were normalized by assigning 0% to the lowest and 100% to the maximum RFU value per individual recording. For PCA, we used the built-in function “prcomp()” in R software (version 3.2.0). The input dataset for PCA comprised of the normalized cellulose optotracing spectral data between 464 and 508 nm from the eight healthy and 182 infected urine samples, as well as from UPEC No. 12 WT and Δ*bcsA* reference samples. PC1 and PC2 were further analyzed with *k*-means clustering, by defining the number of clusters (*k*) with the Elbow method and subsequently using the built-in function “*k*-means()” in R software (version 3.2.0). All plots were prepared using Prism (version 6.0).

## Electronic supplementary material

Supplemental Material

## Data Availability

The authors declare that data supporting the findings of this study are available upon reasonable request.

## Electronic supplementary material

**Supplementary information** accompanies the paper on the *npj Biofilms and Microbiomes* website (10.1038/s41522-018-0069-y).
