# Supplementary material for: Rapid diagnostic assay for detection of cellulose in urine as biomarker for biofilm-related urinary tract infections
Source: NPJ Biofilms Microbiomes. 2018 Oct 26;4:26. doi: 10.1038/s41522-018-0069-y (PMC6203724; doi:10.1038/s41522-018-0069-y)
Supplement: Supplementary file 1 — Supplemental Material [file 41522_2018_69_MOESM1_ESM.pdf]

SUPPLEMENTARY INFORMATION

Rapid diagnostic assay for detection of cellulose in urine as biomarker for biofilm-related urinary tract infections

Haris Antypas[1], Ferdinand X. Choong[1], Ben Libberton[1, #], Annelie Brauner[2, 3], Agneta Richter-Dahlfors[1]*

[1] Swedish Medical Nanoscience Center, Department of Neuroscience, Karolinska Institutet, Stockholm, Sweden.

[2] Department of Microbiology, Tumor and Cell Biology, Karolinska Institutet

[3] Division of Clinical Microbiology, Karolinska University Hospital, Stockholm, Sweden.

[#] Present address: MAX IV Laboratory, Lund University, Lund, Sweden

* Corresponding author

Agneta Richter-Dahlfors
Karolinska Institutet,
Swedish Medical Nanoscience Center
Department of Neuroscience
Solnavägen 9, SE- 171 65
Stockholm, Sweden

Tel: +46 8 5248 7425
email: Agneta.Richter.Dahlfors@ki.se

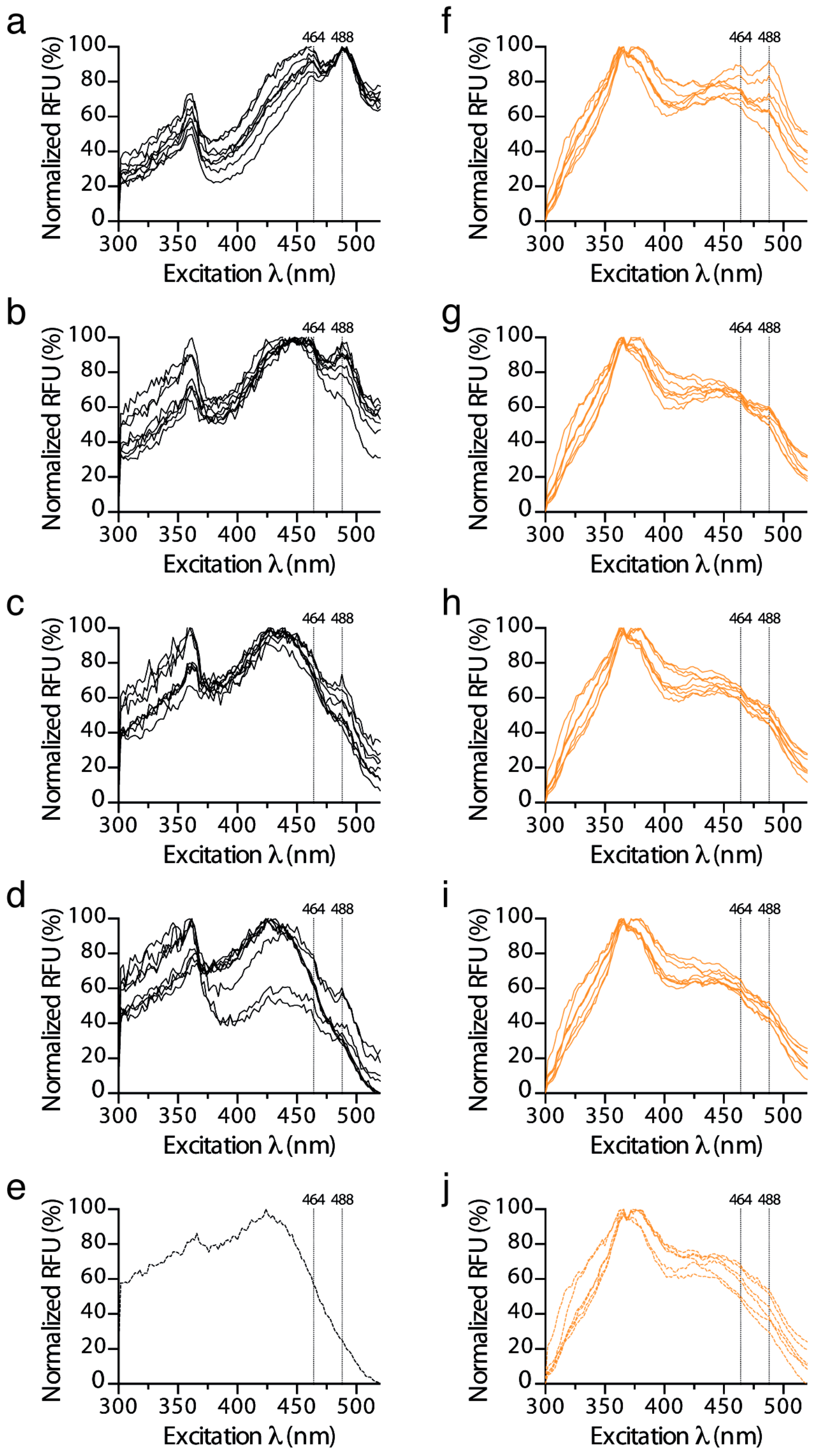

Fig. S1 Normalized optotracing spec-plots from 8 preparations of serial 2-fold dilutions of M. Cellulose in **(a-e)** PBS and **(f-j)** urine. **(a, f)** 62.5 µg/ml, **(b, g)** 31.3 µg/ml, **(c, h)** 15.6 µg/ml, **(d, i)** 7.8 µg/ml, and **(e, j)** 0 µg/ml of M. Cellulose. Each line represents the average normalized fluorescence from 3 technical replicates per preparation. Dotted vertical lines = 464 nm and 488 nm representing the cellulose signature.

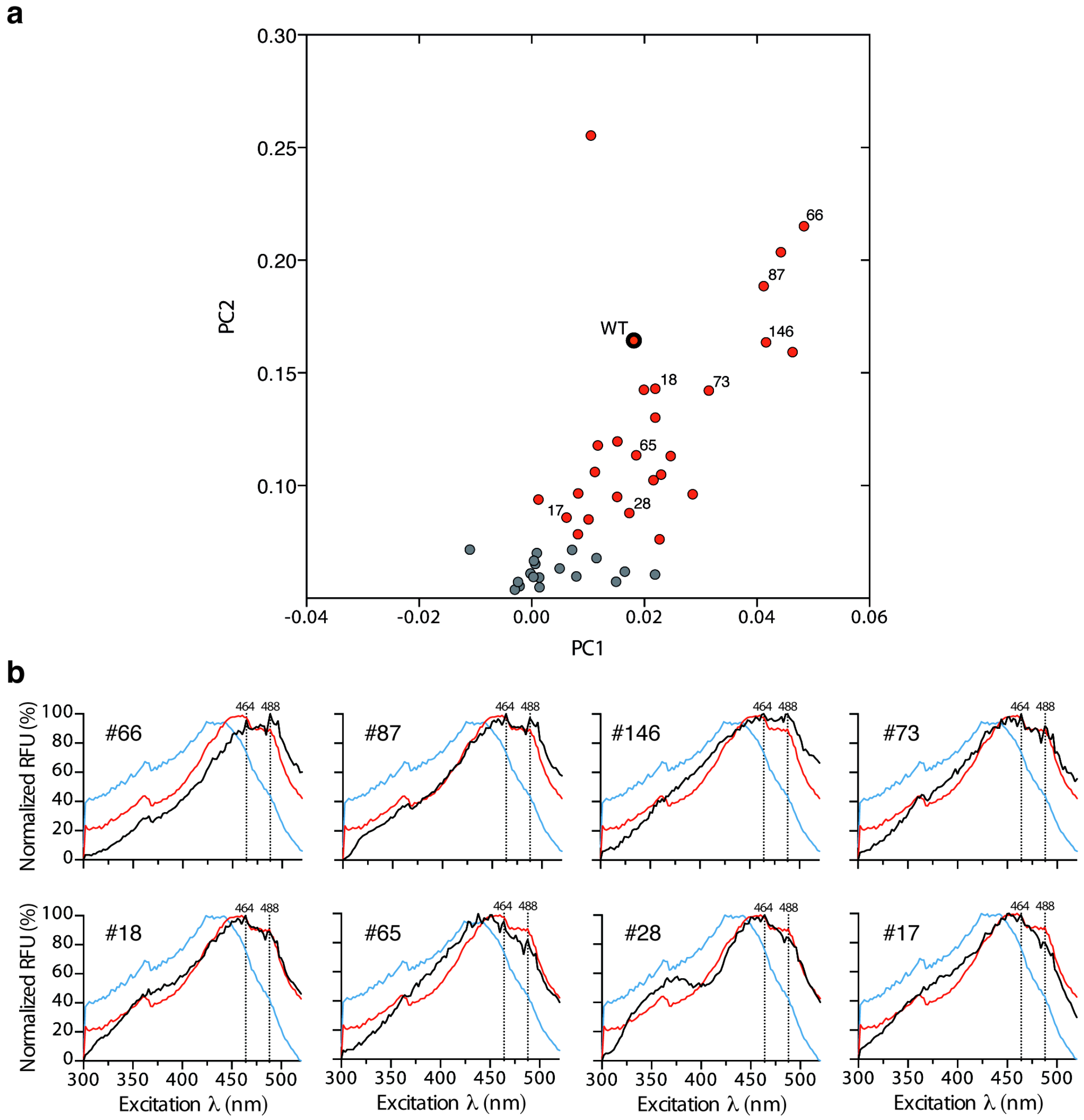

Fig. S2 (a) Magnified area of PCA plot shown in Fig. 3, focusing on cellulose positive urine samples (red circles). Representative samples selected for further analysis are annotated with their ID number and cellulose positive reference is annotated as WT. **(b)** Normalized optotracing spec-plots of representative UTI urine samples (black line). Identification number (#) in the spec-plots corresponds to the annotated samples in **(a).** Normalized optotracing spectra from WT (red line) and Δ*bcsA* (blue line) biofilm are included for comparison. Average normalized fluorescence from 3 technical replicates is shown. Dotted vertical lines = 464 nm and 488 nm representing the cellulose signature.

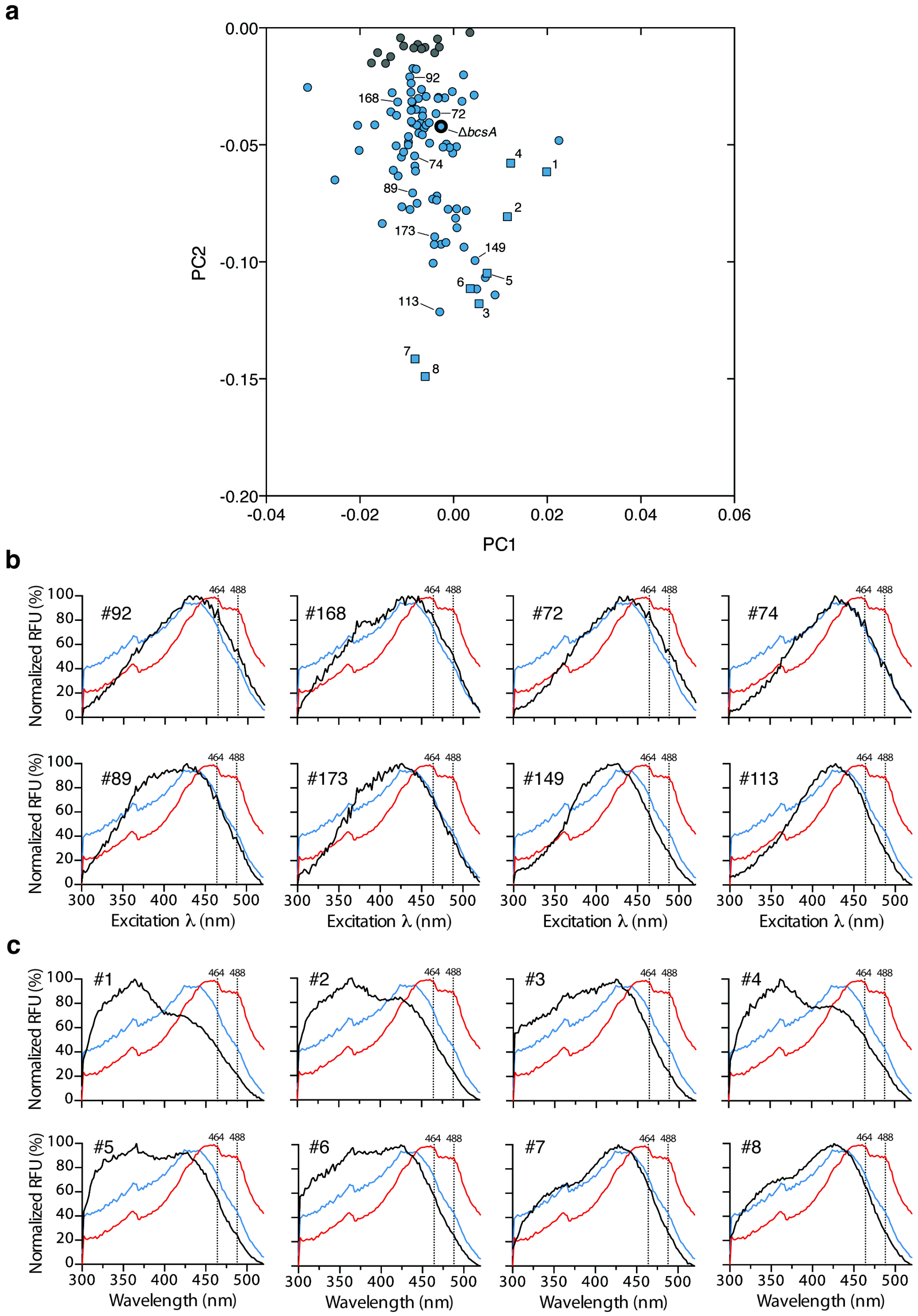

Fig. S3 (a) Magnified area of PCA plot shown in Fig. 3, focusing on cellulose negative urine samples from UTI patients (blue circles) and healthy volunteers (blue squares). Representative samples selected for further analysis are annotated with their ID number and cellulose negative reference is annotated as Δ*bcsA.* **(b-c)** Normalized optotracing spec-plots of representative **(b)** UTI and **(c)** healthy urine samples (black line). Identification number (#) in the spec-plots corresponds to the annotated samples

in **(a).** Normalized optotracing spectra from WT (red line) and Δ*bcsA* (blue line) biofilm are included for comparison. Average normalized fluorescence from 3 technical replicates is shown. Dotted vertical lines = 464 nm and 488 nm representing the cellulose signature.

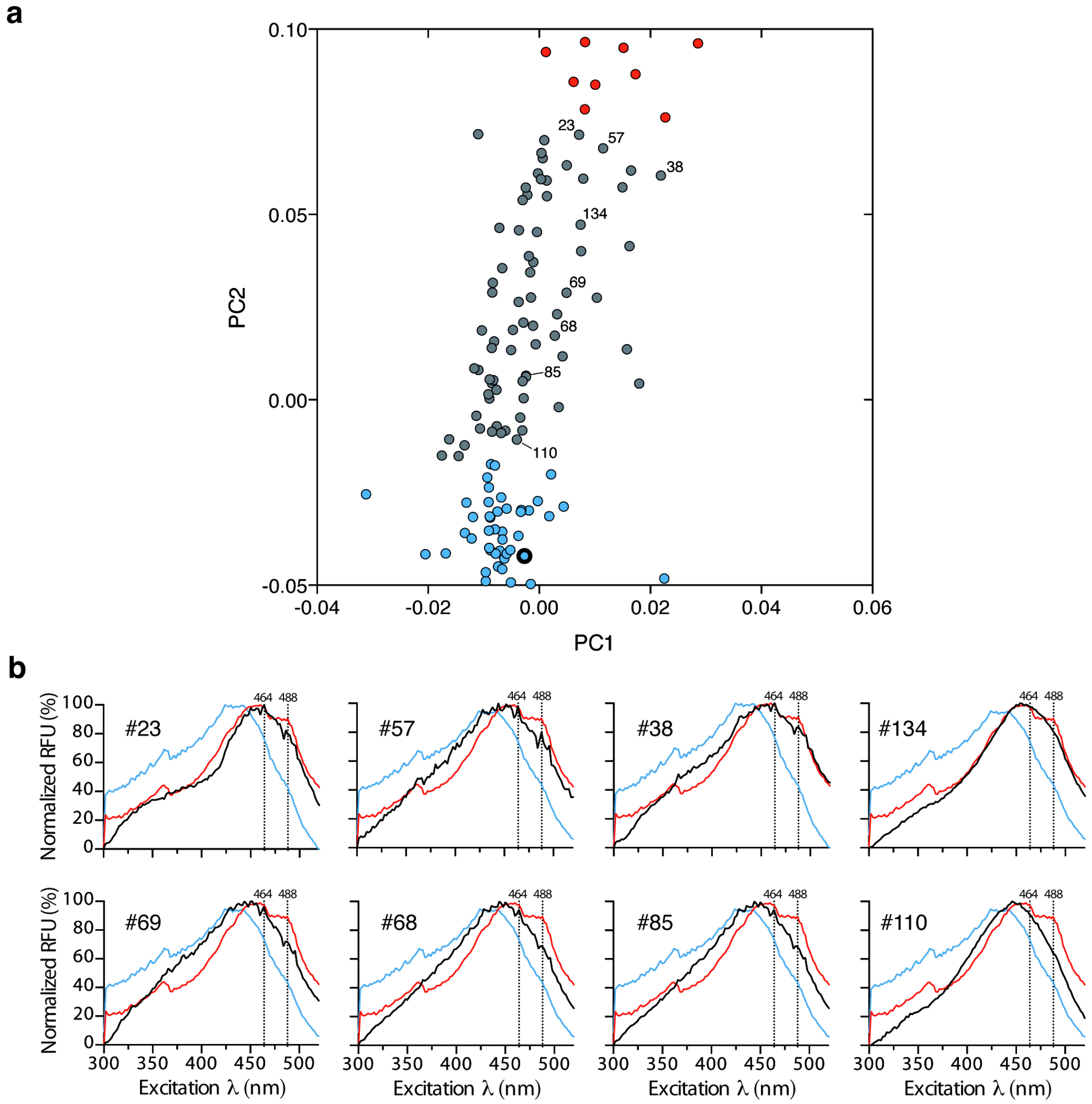

Fig. S4 (a) Magnified area of PCA plot shown in Fig. 3, focusing on urine samples with spectra with insufficient discriminatory performance (grey circles). Representative samples selected for further analysis are annotated with their ID number. **(b)** Normalized optotracing spec-plots of representative UTI urine samples (black line). Identification number (#) in the spec-plots corresponds to the annotated samples in **(a).** Normalized optotracing spectra from WT (red line) and Δ*bcsA* (blue line) biofilm are included for comparison. Average normalized fluorescence from 3 technical replicates is shown. Dotted vertical lines = 464 nm and 488 nm representing the cellulose signature.
